# Low-Power and High-Performance Double-Node-Upset-Tolerant Latch Using Input-Splitting C-Element

**DOI:** 10.3390/s25082435

**Published:** 2025-04-12

**Authors:** Qi Chen, Binyu He, Renjie Kong, Pengjia Qi, Yanyun Dai

**Affiliations:** 1 School of Information Science and Engineering (School of Cyber Science and Technology), Zhejiang Sci-Tech University, Hangzhou 310018, China; 202220702013@mails.zstu.edu.cn (Q.C.); 2023220704022@mail.zstu.edu.cn (B.H.); 2022331200040@mails.zstu.edu.cn (R.K.); qipengjia@zstu.edu.cn (P.Q.); 2 Provincial Key Laboratory for Research and Translation of Kidney Deficiency-Stasis-Turbidity Disease, Hangzhou 310018, China

**Keywords:** double-node-upset, latch, robust, APDP

## Abstract

Data accuracy is critical for sensor systems. As essential components of digital circuits within sensor systems, nanoscale CMOS latches are particularly susceptible to single-node upsets (SNUs) and double-node upsets (DNUs), which can lead to data errors. In this paper, a highly robust Double-Node-Upset-Tolerant Latch-Based on Input Splitting C-Elements (DNUISC) is proposed. The DNUISC latch is designed by interconnecting three sets of input-splitting C-elements to form a feedback loop, and it incorporates clock gating and fast-path techniques to minimize power consumption and delay. Simulations are conducted using the 28 nm process in HSPICE. The simulation results show that the DNUISC can self-recover from any single-node upset and is tolerant of any double-node upset. Compared with existing hardened latches, the DNUISC achieves a 55.21% reduction in area-power-delay product (APDP). Furthermore, the proposed DNUIS demonstrates high reliability and low sensitivity under varying process, voltage, and temperature conditions.

## 1. Introduction

Sensors are increasingly being deployed in healthcare, automotive, industrial, and aerospace applications. At the same time, different application scenarios impose new demands on sensor systems in terms of power consumption and accuracy. According to the proposed AEC-Q100 standard by the Automotive Electronics Council (AEC), electronic devices used in automobiles must operate over a wide temperature range and exhibit high reliability [1]. Moreover, in aerospace applications, the reliability of circuits can significantly decline due to the effects of high-energy radiation in space [2]. In harsh external environments, sensitive nodes may accumulate charges generated by high-energy particle strikes. Once the accumulated charge exceeds the critical charge at the node, it can lead to a transient pulse known as Single Event Transients (SETs) [3]. In sequential elements, these SETs can cause data corruption, resulting in single-event upsets (SEUs) [4,5]. SEUs, also known as soft errors (SEs), can significantly impact the reliability of digital circuits [6].

Latches are indispensable components in the digital circuits of sensor systems, such as ADCs and MCUs, and their performance directly influences overall system stability and accuracy. However, the detrimental impact of SEUs underscores the need to design high-performance latches with robust tolerance capabilities to ensure reliable operation in extreme environments. Radiation-Hardened-by-Design (RHBD) is a circuit-level design technique that provides an efficient solution for improving circuit reliability [7]. Over the years, various RHBD latches have been proposed. However, as technology continues to improve, device density increases and the distance between semiconductor devices decreases. This results in charges collected from single events being able to affect multiple nodes via charge-sharing mechanisms, causing double-node upset (DNU) [8,9]. Traditional single-node upset (SNU) tolerant designs are no longer sufficient to completely prevent DNU. Consequently, soft errors induced by DNU have become increasingly prominent, leading to a rise in the soft error rate within circuits and systems. Although several DNU-tolerant latches have been proposed [10,11,12,13,14,15,16,17], they still exhibit some shortcomings. For instance, some designs fail to achieve SNU self-recovery, or some sacrifice power consumption and delay performance in order to enhance robustness. Furthermore, some designs exhibit high sensitivity to PVT variations.

To address these issues, this paper proposes a Double-Node-Upset-Tolerant Latch-Based Input Splitting C-Element (DNUISC), which effectively balances power consumption and reliability. The features of the proposed latch are listed below. (1) Based on the commonly used C-element (CE) in hardened latch designs, we introduce the Input-Splitting C-element (ISCE) design for the latch [18]. Since the input signals are at the same level during normal operation, the ISCE design can achieve the functionality of a three-input C-element with fewer transistors. Based on ISCE, we propose a latch with SNU self-recovery and DNU tolerance. (2) The use of transmission gates (TG) facilitates the efficient transfer of input signals to output, resulting in a short critical path and minimal delay. Additionally, three TGs are used to transmit input signals to the feedback path as a backup, which enhances the reliability of the circuit. (3) Through careful design, all internal nodes and output nodes of the latch are free from contention, ensuring high stability in performance. The proposed latch demonstrates excellent performance across a wide range of voltage and temperature conditions. (4) Compared with existing DNU-tolerant latches, the proposed DNUISC reduces the area-power-delay product (APDP) by 55.21–89.37%.

The structure of this paper is as follows: Section 2 reviews several advanced DNU-hardened latches. Section 3 describes the circuit structure of the ISCE and explains the working principle and logical validation of the DNUISC latch. Section 4 compares the proposed DNUISC with existing advanced latches in terms of area, power consumption, and delay, with simulation results for different process, voltage, and temperature (PVT) conditions. Section 5 concludes the paper.

## 2. Existing Hardened Latches

In this section, the commonly used techniques in the latches and the hardened latches proposed in recent years are reviewed.

### 2.1. Dual Interlocked Storage Cell (DICE) and C-Element (CE)

**Figure 1:**
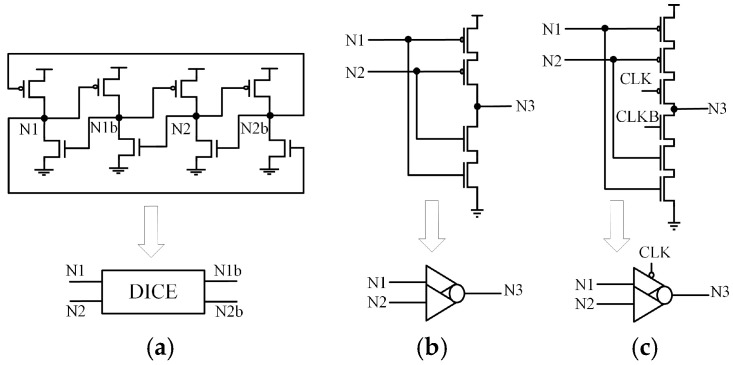
(**a**) DICE. (**b**) Two-input CE. (**c**) CG-based two-input CE.

In hardened latch technology, the Dual Interlocked Storage Cell (DICE) [19] and C-elements (CE) are widely used. Figure 1a illustrates the schematic of a DICE latch. DICE is a cross-coupled structure with four internal nodes that are complementary in pairs. When any node undergoes a reversal, it is corrected by the adjacent node. However, if the values of the adjacent nodes change simultaneously, the stability of the DICE latch is compromised. Additionally, due to circuit contention, circuits employing DICE may experience increased delays.

Figure 1b,c present the schematics and simplified symbols of a two-input CE and a clock-controlled two-input C-element (CG-CE). CE can tolerate single-node upsets (SNU) at its input nodes. When the inputs of CE are the same, the output is the inverse of the input. When the inputs differ, the CE retains its original value and enters a high-impedance state (HIS). However, CE cannot self-recover.

### 2.2. CLCT

**Figure 2:**
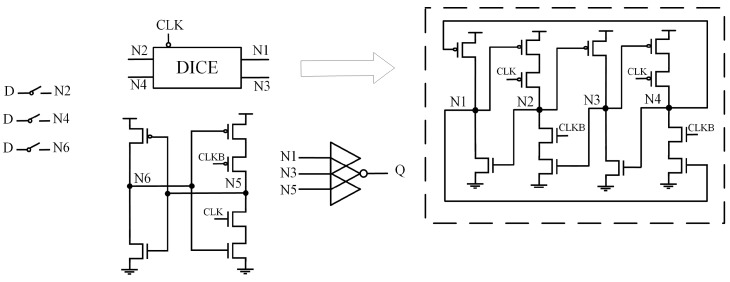
CLCT.

Figure 2 shows the circuit structure of the Multiple-Nodes-Upset Tolerant Latch-Based Circuit and Layout Combination Technique (CLCT) [20]. This latch consists of clock-controlled DICE units and inverters, forming two transmission paths. The three-input CE intercepts the influence of other nodes on the output through its high-impedance state. When errors occur simultaneously in the DICE and inverter paths, the DICE can self-recover from SNU, ensuring the output remains unaffected. However, when the error occurs in the output node and inverter path, the output cannot recover to the correct value. Therefore, CLCT does not provide complete DNU tolerance.

### 2.3. LPDHL

**Figure 3:**
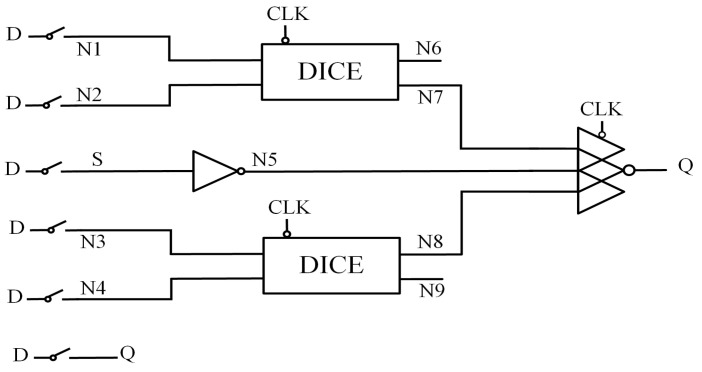
LPDHL.

Figure 3 presents the structure of the Low-Power Double-Node-Upsets Hardened Latch (LPDHL) [10]. This latch mainly utilizes the SNU self-recovery feature of DICE to achieve DNU tolerance. Two inputs of the three-input CG-CE come from two clock-controlled DICE units, while the third input is from a separate inverter. Due to the blocking effect of the CE, both SNU and DNU do not affect the output. When one of the faulty nodes is the output node, the other node is restored by DICE, causing the output to return to its normal value. However, the circuit has specific layout requirements to ensure complete DNU self-recovery because the circuit structure of LPDHL cannot realize the function of node S and Q self-recovery.

### 2.4. DCTELC

**Figure 4:**
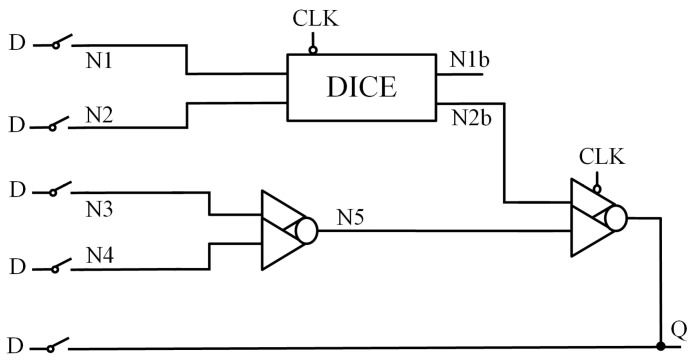
DCTELC.

Figure 4 presents the structure of the Double-Node-Upset Completely Tolerant Latch with Extremely Low Cost (DCTELC) [11]. It consists of a clocked DICE unit (CG-DICE), a two-input CE, a two-input CG-CE, and four transmission gates. The transmission gates effectively reduce the delay from input to output. The CG-DICE can self-recover from internal SNU and avoid power consumption due to circuit contention. Another path, consisting of CG-CE and CE, is also SNU-tolerant. In this circuit, DNU affects only one path while the other maintains the correct value, ensuring that the output signal remains unaffected. Therefore, DCTELC can fully tolerate double-node upsets. However, affected by the characteristics of the CE, nodes N3 and N4 cannot achieve SNU self-recovery.

### 2.5. CDNUTC

**Figure 5:**
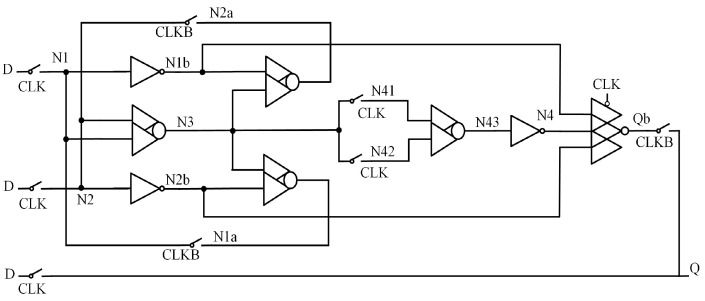
CDNUTC.

Figure 5 shows the structure of the Latch Design with Complete Double-Node-Upset Tolerant Capability Using C-Element (CDNUTC) [12]. This latch is primarily composed of transmission gates (TG), two-input CE, three-input CE, and inverters. The feedback structure formed by the two-input CE and transmission gate enables partial node SNU self-recovery. To achieve full DNU tolerance, transmission gates divide the circuit into two parts, which are then combined using a three-input CE. The use of clock-gating technology effectively reduces both power consumption and delay. However, the circuit is divided into two parts, and there is no feedback loop in the second half, so some nodes cannot achieve SNU self-recovery.

### 2.6. HRDNUT

**Figure 6:**
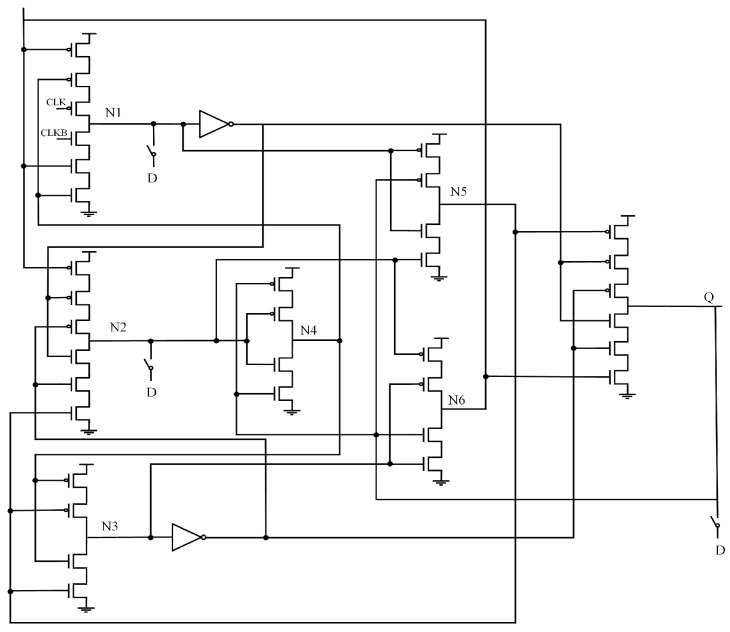
HRDNUT.

Figure 6 presents the circuit structure of the Highly Robust Double-Node-Upset Tolerant latch (HRDNUT) [13]. This latch consists of a two-input CE, a two-input CG-CE, a three-input CE, and inverters, forming a three-stage feedback loop. Since SNU cannot affect the output of the CE, the three-stage feedback loop allows for complete SNU self-recovery. Clock-gating technology and transmission gates reduce power consumption from circuit contention and shorten the input-to-output path, reducing delay. However, the output signal is involved in internal feedback, and the latch is not entirely free from contention, leading to increased power consumption and delay.

### 2.7. LSEDUT

**Figure 7:**
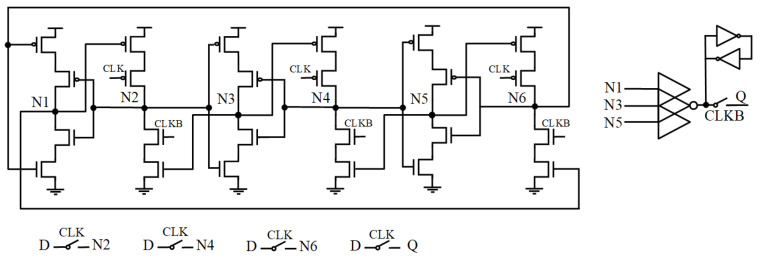
LSEDUT.

Figure 7 shows the circuit diagram of the Low-Cost Single Event Double-Upset Tolerant Latch (LSEDUT) [14]. This latch uses interlocked nodes in the storage unit to retain data and employs clock-gated CE at the data transmission nodes to disable the storage unit. The weak keeper at the output node prevents high-impedance states from affecting the output. Transmission gates significantly reduce transmission delay while eliminating power loss caused by current contention. The three-input CE blocks soft errors from the storage unit, ensuring that the output is correct. Thus, this circuit can fully tolerate both SNU and DNU. However, the keeper generates circuit contention, which increases the power consumption and delay of the circuit.

### 2.8. DNUSH

**Figure 8:**
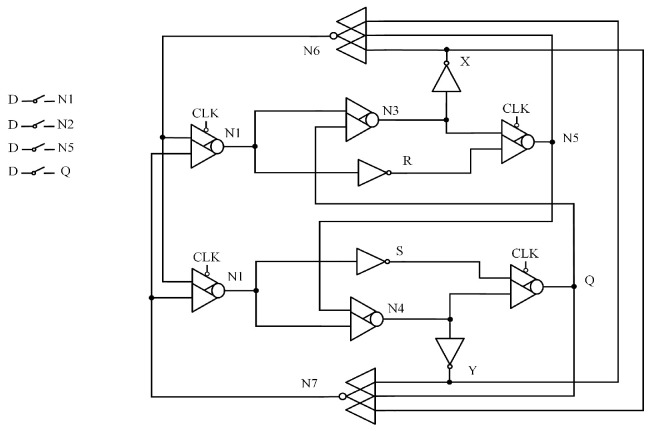
DNUSH.

The Double Node Upset Self-Healing Latch (DNUSH) [15] shown in Figure 8 comprises two identical redundant modules. Each module utilizes multiple CEs and inverters to form feedback loops, achieving complete tolerance to DNU. Additionally, the inclusion of CG-CE ensures that the latch operates without contention during transmission modes, thereby reducing power consumption. However, the extensive use of CEs and CG-CEs increases the latch’s area and power overhead. In addition, the participation of the output signal Q in the feedback will affect the delay.

### 2.9. LCDRL

**Figure 9:**
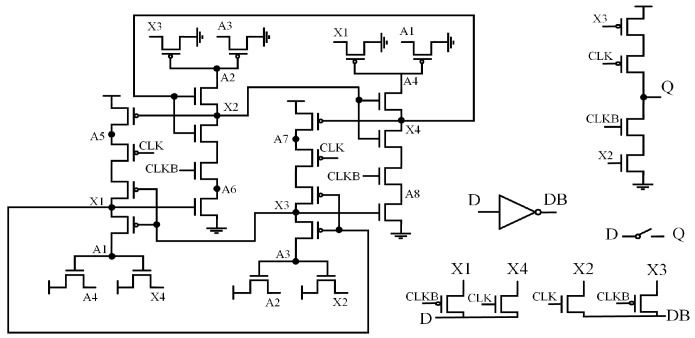
LCDRL.

Figure 9 displays the Low-Complexity Double-Node-Upset Resilient Latch (LCDRL) [16] design, which introduces a novel dual-module redundancy architecture. Unlike the DICE principle, LCDRL aims to minimize redundancy. It does not require feedback paths to restore corrupted data, as one latch module is SEU-insensitive while the other is sensitive. This design significantly reduces the transistor count associated with multiple redundant modules. However, LCDRL imposes stricter timing requirements on the circuit. To meet functional demands, adjustments in transistor sizes and the use of high-threshold transistors are required, resulting in increased power consumption and additional area overhead.

### 2.10. DRLW

**Figure 10:**
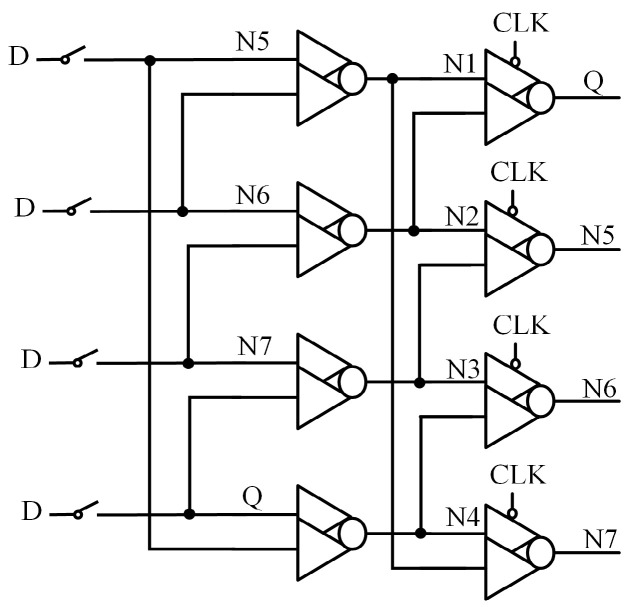
DRLW.

Figure 10 illustrates the circuit structure of the Double-Node-Upset Self-Recoverable Latch for Wide Voltage Range Application (DRLW) [17]. This latch consists of four transmission gates (TGs), four two-input CEs, and four two-input CG-CEs, organized into two feedback loops. By connecting CEs and CG- CEs in series to form feedback loops, the latch achieves complete DNU tolerance, and clock gating technology reduces power consumption. However, due to the influence of the feedback circuit, the circuit delay will be larger.

## 3. Proposed Hardened Latch Design

This section primarily describes the circuit structure of the Input Splitting C-element (ISCE) and the circuit design and operating principles of the proposed DNUISC latch. Additionally, the logical functionality of the DNUISC is validated through fault injection.

### 3.1. Working Principle of Input Splitting C-Element

**Figure 11:**
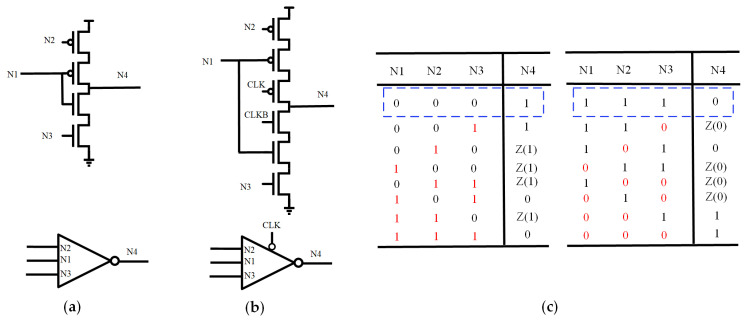
(**a**) The input splitting CE. (**b**) The Clock-Gated input splitting CE. (**c**) State table.

Figure 11 shows the circuit structure of ISCE, clock-gated ISCE (CG-ISCE), and the state table of ISCE, respectively. In this circuit, N1 serves as the intermediate input node, while N2 and N3 correspond to the PMOS and NMOS input nodes, respectively. Under normal conditions, the logic states of input nodes N1, N2, and N3 are identical, with the output node N4 being the inverse of these inputs.

The dashed-boxed region in the state table represents the correct initial values, while the red section indicates the state after a flip occurs, with “Z” denoting a high-impedance state. As shown in Figure 11c, if the initial state of <N1, N2, N3, N4> is <0, 0, 0, 1>, a simultaneous flip of N1 and N3 will cause the output N4 to flip. Similarly, if the initial state of <N1, N2, N3, N4> is <1, 1, 1, 0>, a simultaneous flip of N1 and N2 will result in a flip of output N4. In all other cases, output N4 remains unchanged from its initial state.

### 3.2. Proposed DNU Tolerant Latch Design

#### 3.2.1. Circuit Structure

**Figure 12:**
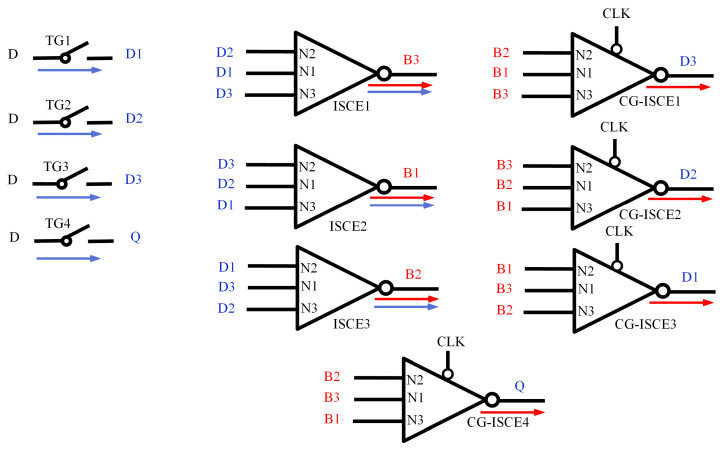
The proposed DNU-tolerant latch.

The DNUISC latch circuit, as depicted in Figure 12, comprises four transmission gates (TG1–TG4), three ISCEs, and four CG-ISCEs, resulting in a total of seven nodes labeled D1–D3, B1–B3, and Q. In this circuit, D represents the input signal, Q denotes the output signal, CLK is the system clock signal, and CLKB is the inverted clock signal. The inputs to the three ISCEs are D1–D3, with outputs B1–B3. The N1 input nodes of these ISCEs are connected to D1, D2, and D3, respectively; the N2 input nodes to D2, D3, and D1; and the N3 input nodes to D3, D1, and D2, respectively. The outputs N4 correspond to the N3 input signals of each ISCE. The four CG-ISCEs receive inputs B1–B3 and produce outputs D1–D3 and Q. The input nodes of CG-ISCE1-3 correspond to those of ISCE1-3, with outputs ordered as D3, D2, and D1. CG-ISCE4 outputs Q, with its N1, N2, and N3 input nodes connected to B3, B2, and B1, respectively.

This arrangement ensures that the inputs to each ISCE are distinct, allowing for the effective utilization of the ISCE’s fault-tolerant properties, where a fault at either the N1 or N2 node does not affect the output, thereby maintaining the correct output signal. ISCE1–3 and CG–ISCE1-3 form feedback loops to achieve complete SNU and partial DNU self-recovery. Additionally, fast-path techniques are employed between the input and output to reduce delay, while clocked transistors ensure that the circuit remains free from current contention during data transmission modes. These design choices effectively minimize delay and power consumption.

#### 3.2.2. Working Principle

When CLK = 1 and CLKB = 0, the DNUISC operates in data transmission mode. The circuit conduction path is shown by the red arrow in Figure 12. In this state, transmission gates TG1–TG4 are conductive, and D1–D3, along with the output signal Q, vary in accordance with the input signal D. Simultaneously, ISCE1–ISCE3 are conductive, so B1–B3 change in response to D1–D3. When CLK = 0 and CLKB = 1, the latch enters hold mode. The circuit conduction path is shown by the blue arrow in Figure 12. In this mode, TG1–TG4 are non-conductive, but ISCE1–ISCE3 and CG-ISCE1–4 remain conductive, thereby maintaining the states of D1–D3, B1–B3, and Q.

#### 3.2.3. Hardening Principle

The DNUISC is designed to tolerate SNU and DNU, with self-recovery capabilities for SNU. Table 1 shows the simplified process. The fault-tolerant mechanisms of DNUISC are as follows:

For SNU, there are three scenarios. (1) The flipped node is located in the D1–D3. Since an ISCE’s output is affected only when both inputs experience faults, B1–B3 and Q remain unaffected. The faulty D node is restored to its correct value by ISCE1–3. (2) The flipped node is located in the B1–B3. This situation mirrors the first one, with the faulty B node being corrected by CG-ISCE3). The flipped node is Q. In this case, D1–D3 and B1–B3 are unaffected by the fault in Q. Subsequently, Q is restored to its correct value by CG-ISCE4.

The DNU fault-tolerant mechanism of the circuit encompasses four scenarios:

(1) Q Flips, and One of D1–D3 or B1–B3 Flips: For instance, if <Q, D1> flips, the other nodes remain unaffected. From the SNU fault-tolerant analysis, D1 is restored by CG-ISCE3, and Q is then corrected by CG-ISCE4.
(2) Two B Nodes Flip: There are two sub-cases:
  Q Flips and Self-Recovers: For example, when <B2, B3> changes from <1, 1> to <0, 0>, Q flips. CG-ISCE2 and CG-ISCE4’s pull-up circuits conduct, causing D2 and Q to change from 0 to 1. ISCE3 remains unaffected by D2. ISCE3’s pull-up circuit conducts, restoring B2 to 1. Then, CG-ISCE2’s pull-down circuit conducts, bringing D2 back to 0. ISCE1’s pull-up circuit restores B3 to 1. Consequently, D1–D3 and B1–B3 are all restored to their correct values, and Q is corrected by CG-ISCE4. A similar process occurs for <B1, B3>.
  Q Remains Unaffected: For example, when <B1, B2> changes from <1, 1> to <0, 0>, CG-ISCE1’s pull-up circuit conducts, causing D2 to change from 0 to 1. Due to CG-ISCE4’s interception, the output Q remains unaffected. In all other cases, Q does not flip.
(3) Two D Nodes Flip: For instance, if <D1, D2> changes from <1, 1> to <0, 0>, ISCE1’s pull-up circuit conducts, causing B3 to change from 0 to 1. Due to CG-ISCE4’s interception, Q remains unaffected. The mechanisms for <D1, D3> and <D2, D3> are analogous. Additionally, when <D2, D3> flips, self-recovery is achieved through the feedback loops of ISCE1–3 and CG-ISCE1–3.
(4) One D Node and One B Node Flip: Since an ISCE’s output is affected only when both inputs experience faults, the other nodes remain unaffected. Therefore, Q remains unchanged.

### 3.3. Fault Injection Experiment

To validate the functionality of the proposed DNUISC latch, fault injection experiments were conducted using HSPICE simulations based on the 28 nm CMOS process. To minimize delay, the PMOS transistor in the transmission gate TG4 was sized with a width-to-length ratio (W/L) of 200 nm/30 nm, while the remaining PMOS and NMOS transistors were sized at the minimum width and channel length (W/L = 100 nm/30 nm). The supply voltage was set at 0.9 V, with a clock frequency of 1 GHz and a temperature of 25 °C. Fault injections were performed during the latch’s hold mode (i.e., when CLK = 0) to isolate the effects from input signal variations. The fault injection nodes were selected based on their susceptibility to faults, including both internal and output nodes [21]. A double-exponential current source model was employed to simulate the transient pulses generated by high-energy particle strikes [22]. The equation for pulse *I*(*t*) is given by (1).

(1) $$I\left(t\right)=\{\begin{array}{c} \;\;\;\;\;\;\;\;\;\;\;\;\;\;\;\;\;\;\;\;\;\;0;\;\;\;\;\;\;\;\;\;\;\;\;\;\;\;\;\;\;\;\;t<\;\;t_{d1}\\ I_{0}\left[1-exp\left(-\frac{t-t_{d1}}{\tau_{1}}\right)\right];\;\;\;\;t_{d1}<t<\;\;t_{d2}\\ I_{0}\left[\exp\left(-\frac{t-t_{d2}}{\tau_{2}}\right)-exp\left(-\frac{t-t_{d1}}{\tau_{1}}\right)\right];\;t>\;\;t_{d2} \end{array}$$

where *I*(*t*) represents the injected transient current, *I*_0_ denotes peak current, *t_d_*_1_ and *t_d_*_2_ represent starting points of the current rise and fall, and *τ*_i_ and *τ*_2_ represent the time constant for the rise and fall, respectively. The parameters *τ*_i_ and *τ*_2_ are set to 2 ps and 10 ps, respectively, as specified in [22].

**Figure 13:**
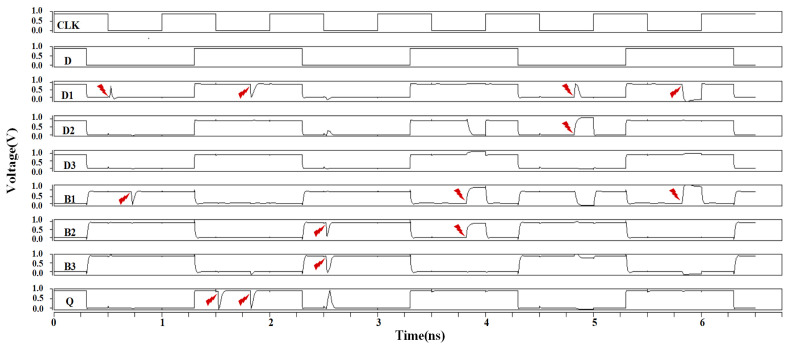
Simulation results of injection into the DNUISE latch.

The simulation results for the DNUISC latch are shown in Figure 13. The red arrows indicate that fault injections are performed. For SNU injections, the node voltages rapidly recovered to their correct values. In the case of DNU fault injections, the output Q remained unaffected or quickly returned to its original value. These observations indicate that the proposed DNUISC latch successfully performs its intended logical functions.

## 4. Simulation Results

In this section, we compare the performance of the proposed DNUISC latch with nine other latches. To ensure fairness, all latches were simulated using a 28 nm process in HSPICE, with a supply voltage of 0.9 V, a frequency of 1 GHz, and a temperature of 25 °C. The PMOS and NMOS transistors were adjusted from their minimum width and channel length (W/L = 100 nm/30 nm) to meet the circuit’s functional requirements.

### 4.1. Performance Comparison of Latches

**Figure 14:**
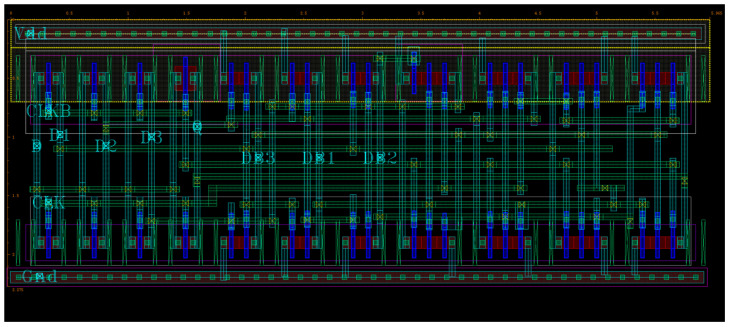
Layout of DNUISC latch.

Table 2 presents a comparison of the latches’ hardening capabilities, power consumption, delay, area overhead, and area-power-delay product (APDP). Delay is defined as the time from the rising edge of the input signal (D) to the output signal (Q), with the average of the rising and falling delays taken. Power refers to the average power consumption of the entire circuit within 18 ns. The layout of DNUISC is shown in Figure 14. Area is measured as the effective area of the latch, as determined by Equation (2) [23,24]. The soft error rate (SER) is calculated using the model of [23,25]. The APDP is used to evaluate overall performance. A smaller APDP indicates better performance [26]. SNUT, SNUR, and DNUT indicate the latch’s hardening capabilities.

(2) $$Area=\overset{n}{\underset{i=1}{\sum }}0.81\left(L_{P}\left(i\right)*W_{P}\left(i\right)+L_{N}\left(i\right)*W_{N}\left(i\right)\right)$$

From the table, it is evident that the proposed latch offers significant advantages in power consumption and delay. In the design of the proposed latch, a significant number of clock-controlled transistors are employed. This design choice increases the area overhead; however, it effectively eliminates circuit contention and offers substantial benefits in terms of power consumption. Additionally, when the output signal participates in internal feedback loops, its delay is susceptible to variations in internal nodes. To mitigate this issue, we utilize a dedicated CG-ISCE4 to control the output signal. Although this approach adds to the area overhead, it significantly reduces delay, ultimately achieving superior overall performance metrics, particularly in terms of APDP. Additionally, the proposed latch is robust, achieving SNU self-recovery and DNU tolerance, and has a low probability of SER. In contrast, latches CLCT, LPDHL, DCTELC, and CDNUTC cannot achieve SNU self-recovery, and latch CLCT cannot achieve DNU tolerance.

For the more intuitive comparison, we calculated the power, delay, area, and APDP of the proposed latch and other latches using Formula (3). The results are presented in Table 3.

(3) $$\Delta =\left(\frac{Compared-Latch-Proposed-Latch}{Compared-Latch}\right)*100\%$$

The data in the table demonstrate that the DNUISC latch exhibits superior performance. It achieves a 13.48–89.89% reduction in power consumption, a 17.92–65.75% reduction in delay, and a 55.21–89.37% improvement in APDP. The latches compared here are those that can achieve SNU self-recovery and DNU tolerance. Therefore, compared with other latches, the DNUISC latch offers lower cost and enhanced performance.

### 4.2. Effect of PVT Variations on Latches

As transistor feature sizes decrease, digital circuits become increasingly sensitive to Process, Voltage, and Temperature (PVT) variations [27]. To further ensure the robustness of the proposed DNUISC latch, this section compares the impact of PVT variations on several latches.

**Figure 15:**
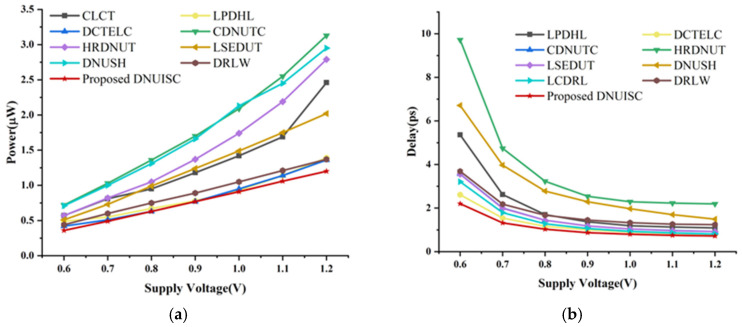
(**a**) Delay at different voltages, (**b**) Power at different voltages.

In energy-constrained devices, designers often use lower supply voltages to reduce circuit power consumption. However, this design choice increases delays and decreases the capacitor charge of circuit nodes [28]. As a result, latches are more susceptible to interference from high-energy particle impacts, causing DNU. Figure 15 presents the power consumption and delay of the latches across a supply voltage range of 0.6 V to 1.2 V. Due to the significant delay in CLCT and the significant power consumption in LCDRL, these parameters are excluded from the figure to maintain the accuracy of other data. The figure shows that power consumption increases with supply voltage while delay decreases. The proposed latch consistently demonstrates the lowest power consumption and delay across the 0.6 V to 1.2 V voltage range. Additionally, the DNUISC latch exhibits relatively stable power consumption and minimal delay variation.

**Figure 16:**
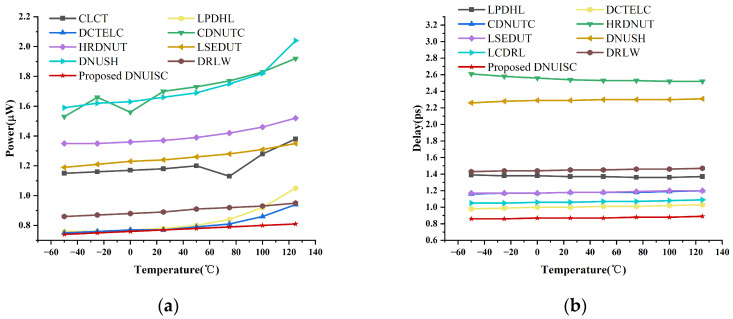
(**a**) Delay at different temperatures. (**b**) Power at different temperatures.

Figure 16 provides two graphs depicting the power consumption and delay of the latches as functions of temperature, ranging from −50 °C to 125 °C. The graph indicates that both power consumption and delay increase slightly with rising temperature. However, these changes are minimal, further confirming the excellent robustness of the proposed latch.

Table 4 presents the performance of the proposed latch under eight different Process, Voltage, and Temperature (PVT) conditions. The abbreviations used are as follows: WC denotes Worst Case, WCH denotes Worst Case Hot, BCF denotes Best Case Fast, and BCS denotes Best Case Slow. SS and FF refer to different process corners, with voltage variations set at 10% and 50%, respectively. Extreme temperatures of −50 °C and 125 °C are also considered.

It is noteworthy that the latch requires the propagation of input signal values to all internal nodes to function correctly. Therefore, PVT variations can influence the operating frequency. At a supply voltage of 0.45 V, which is below the normal operating range, the latch’s performance at a 1 GHz operating frequency is suboptimal. To ensure reliable operation, we have adopted a 10 MHz operating frequency. Among the eight PVT scenarios, the low-temperature, low-voltage condition with the SS process corner exhibits the worst-case performance due to temperature inversion effects.

**Figure 17:**
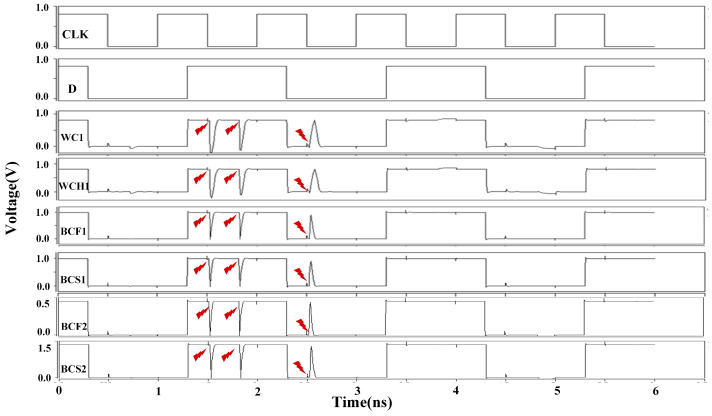
Simulation results of injection under extreme PVT conditions (1 GHz).

**Figure 18:**
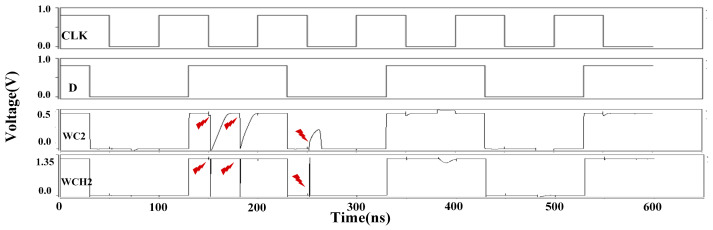
Simulation results of injection under extreme PVT conditions (10 MHz).

Figure 17 and Figure 18 illustrate the results of fault injection simulations under various PVT conditions at clock frequencies of 1 GHz and 10 MHz, respectively. The results demonstrate that the DNUISC latch maintains robust functionality even under these extreme conditions. Specifically, at a supply voltage of 0.45 V, the latch consumes minimal power, making it suitable for energy-constrained applications. In contrast, at a supply voltage of 1.35 V, the latch achieves low latency, making it suitable for high-speed applications.

## 5. Conclusions

In this paper, we propose the DNUISC latch, which utilizes three interconnected ISCE and CG-ISCE units to form a feedback loop, with a CG-ISCE unit providing the output signal. The latch employs clock gating and fast path techniques to minimize power consumption and delay. HSPICE simulation results demonstrate that the DNUISC performs as intended, effectively recovering from all SNUs and tolerating all DNUs. Compared with existing DNU-tolerant latches, it offers superior performance in terms of power consumption, delay, and APDP. Additionally, the DNUISC exhibits high reliability with low sensitivity to PVT variations. Currently, we are actively exploring the development of DNU and TNU self-restoring latches using Input-Split C-Element (ISCE) units. Implementing these self-restoring latches is expected to incur a larger area overhead.

## Figures and Tables

**Table 1:** Processes of typical SNU and DNU hardening principles.

Status	Fault	Process	Result
D = Q = 0	D1: 0→1	D1(CG-ISCE3): 1→0	D = Q = 0
D = Q = 0	B1: 1→0	B1(ISCE2): 0→1	D = Q = 0
D = Q = 1	Q: 1→0	Q(CG-ISCE4): 0→1	D = Q = 1
D = Q = 1	D1: 1→0; Q: 1→0	D1(CG-ISCE3): 0→1; Q(CG-ISCE4): 0→1	D = Q = 1
D = Q = 0	B2: 1→0; B3: 1→0	Q(CG-ISCE4): 0→1; D2(CG-ISCE2): 0→1; B2(ISCE3): 0→1; D2(CG-ISCE2): 1→0; B3(ISCE1): 0→1; Q(CG-ISCE4): 1→0	D = Q = 0
D = Q = 1	B1: 0→1; B2: 0→1	D2(CG-ISCE2): 1→0	D = Q = 1
D = Q = 0	D1: 1→0; D2: 1→0	B3(ISCE1): 0→1	D = Q = 0
D = Q = 1	D1: 1→0; B1: 0→1	D1(CG-ISCE3): 0→1; B1(ISCE2): 1→0	D = Q = 1

**Table 2:** Performance comparison results of latches.

Latches	Power (μW)	Delay (ps)	Area (10^−5^ × nm^2^)	SER (10^−2^ × A.U.)	APDP (10^−5^×)	Hardener Type		
						SNUT	SNUR	DNUT
CLCT [20]	1.18	21.68	1.00	44.65	25.58	YES	NO	NO
LPDHL [10]	0.78	1.37	1.12	11.52	1.20	YES	NO	YES
DCTELC [11]	0.77	1.00	0.97	11.28	0.75	YES	NO	YES
CDNUTC [12]	1.7	1.18	1.02	10.30	2.05	YES	NO	YES
HRDNUT [13]	1.37	2.54	1.07	10.73	3.72	YES	YES	YES
LSEDUT [14]	1.24	1.18	1.26	10.57	1.84	YES	YES	YES
DHUSH [15]	1.66	2.29	1.45	14.18	5.51	YES	YES	YES
LCDRL [16]	4.95	1.06	1.31	12.55	6.87	YES	YES	YES
DRLW [17]	0.89	1.45	1.26	11.55	1.63	YES	YES	YES
Proposed Latch	0.77	0.87	1.09	10.54	0.73	YES	YES	YES

**Table 3:** Relative changes in performance.

Latches	ΔPower	ΔDelay	ΔArea	ΔAPDP
HRDNUT [13]	43.8%	65.75%	−1.87%	80.38%
LSEDUT [14]	37.9%	26.27%	13.49%	60.33%
DHUSH [15]	53.61%	62.01%	24.83%	86.75%
LCDRL [16]	89.89%	17.92%	16.79%	89.37%
DRLW [17]	13.48%	40%	13.49%	55.21%

**Table 4:** Simulation results of extreme PVT conditions.

Name	Corner	Voltage (V)	Temperature (°C)	Frequency (Hz)	Power (nW)	Delay (ps)	SER (10^−2^ × A.U.)
WC1	SS	0.81	−50	1G	605.40	1.50	10.73
WCH1	SS	0.81	125	1G	675.52	1.39	10.71
BCF1	FF	0.99	−50	1G	813.52	0.61	10.42
BCS1	FF	0.99	125	1G	996.17	0.65	10.41
WC2	SS	0.45	−50	10M	1.80	87.17	11.01
WCH2	SS	0.45	125	10M	5.07	7.78	10.97
BCF2	FF	1.35	−50	1G	1514.2	0.49	10.04
BCS2	FF	1.35	125	1G	1790.3	0.57	9.98

## Data Availability

Data are contained within the article.
